# Structure–Function Analysis Reveals the Singularity of Plant Mitochondrial DNA Replication Components: A Mosaic and Redundant System

**DOI:** 10.3390/plants8120533

**Published:** 2019-11-21

**Authors:** Luis Gabriel Brieba

**Affiliations:** Laboratorio Nacional de Genómica para la Biodiversidad, Centro de Investigación y de Estudios Avanzados del IPN, Apartado Postal 629, Irapuato, Guanajuato C.P. 36821, Mexico; luis.brieba@cinvestav.mx

**Keywords:** DNA replication, evolution, replisome, recombination-dependent replication

## Abstract

Plants are sessile organisms, and their DNA is particularly exposed to damaging agents. The integrity of plant mitochondrial and plastid genomes is necessary for cell survival. During evolution, plants have evolved mechanisms to replicate their mitochondrial genomes while minimizing the effects of DNA damaging agents. The recombinogenic character of plant mitochondrial DNA, absence of defined origins of replication, and its linear structure suggest that mitochondrial DNA replication is achieved by a recombination-dependent replication mechanism. Here, I review the mitochondrial proteins possibly involved in mitochondrial DNA replication from a structural point of view. A revision of these proteins supports the idea that mitochondrial DNA replication could be replicated by several processes. The analysis indicates that DNA replication in plant mitochondria could be achieved by a recombination-dependent replication mechanism, but also by a replisome in which primers are synthesized by three different enzymes: Mitochondrial RNA polymerase, Primase-Helicase, and Primase-Polymerase. The recombination-dependent replication model and primers synthesized by the Primase-Polymerase may be responsible for the presence of genomic rearrangements in plant mitochondria.

## 1. Introduction

### 1.1. Plant Mitochondria Genomes

Mitochondria arose from a monophyletic endosymbiotic event between an archaea and an α-proteobacteria approximately two billion years ago [1]. During the evolution of eukaryotes, mitochondrial genomes have evolved in size and complexity. For instance, mitochondrial genomes vary in size more than three orders of magnitude and they exist as circular, linear, linear-branched, linear-fragmented, and mixtures of maxi and mini-circles [2]. In general, metazoan mitochondrial genomes are circular molecules that vary in sizes between 10 to 30 kb [3]. In contrast, plant mitochondrial genomes are predominantly large linear DNA molecules (up to 11 Mb in angiosperms from the genus Silene). Besides the differences between the physical structure of the plant and metazoan genomes (linear versus circular), the most remarkable characteristics of plant mitochondrial genomes are their ability to rearrange, their low nucleotide substitution rate, and the evolution of new mitochondrial open reading frames. For instance, almost all vertebrates exhibit a similar organization in their mitochondrial genome arrangement [4], whereas the mitochondrial genomic organization in plants is different even between ecotytpes of the same species [5]. The abundance of noncoding sequences severely complicates alignments of mitochondrial genomes from different plant families [6]. A comparison between the mitochondrial genomes of Col-0 and C24 ecotypes of *Arabidopsis thaliana*, that diverged 200,000 years ago, shows that both genomes exhibit different configurations because of a large inverted repeat [5,7,8,9]. Even though plant mitochondrial genomes rearrange, the substitution rate in their coding regions is almost negligible, in contrast with the highly mutable human mitochondrial genome [10,11].

### 1.2. Replication in Mammalian Mitochondria

Due to their bacterial origin, the mechanisms involved in mitochondrial and plastid DNA replication are expected to be related to bacteria. Yet mitochondrial DNA replication in metazoans is achieved by a replisome that is phylogenetically related to the bacteriophage T7 replisome [12,13]. In mitochondrial replisomes from metazoans, a bacteriophage-related RNA polymerase synthesizes RNA primers to start replication at the heavy and light chains of the circular DNA mitochondrial molecule, a hexameric helicase unwinds double-stranded DNA, and a trailing mitochondrial DNA polymerase synthesizes DNA. Human mitochondrial DNA replication starts by a strand-displacement model of replication in which human mitochondrial RNA polymerase (RNAP) transcribes the heavy-strand promoter generating a primer that is processed and passed on to the mitochondrial DNA polymerase (DNAP), DNA replication proceeds interruptedly to copy a new heavy-strand [14]. During this process, the replication fork replicates the light strand origin of replication. This DNA sequence folds into a stem–loop structure that allows primer synthesis by the mitochondrial RNAP, and these primers are elongated by the mitochondrial DNA polymerase [15]. Elongation of the heavy and light chains continues asynchronically until the two chains are completely copied. Although the strand-displacement model is generally accepted as the mechanism for mitochondrial DNA replication, there are discrepancies regarding how it proceeds. To date, two alternative models explain strand-asynchronous replication in mitochondria. One model proposes that long RNA molecules hybridize to the single-stranded heavy-strand [16]. This ribonucleotide (RNA) incorporation occurred throughout the lagging strand (RITOLS) transcripts that are continuously hybridized as replication continues [17]. The second model proposes that single-stranded DNA binding proteins coat the lagging-strand template [18]. Alternatively to the strand-displacement model, coupled leading and lagging-strand DNA synthesis can occur bidirectionally in mitochondria [19,20] and recent work stablished that cells can shift between the strand-asynchronous and the coupled leading and lagging-strand DNA synthesis depending of the amount of transcripts [21].

## 2. Enzymes Involved in Organelle DNA Replication in Plants Can Be Grouped into Bacteriophage-Related, Replication-Dependent Replication and Unique Enzymes

The main difference between the mitochondrial metazoan and bacteriophage T7 replisomes is that the T7 primase-helicase harbors an active primase module that synthesizes primers for lagging strand synthesis, whereas the primase module of metazoan primase-helicases is inactive and primer synthesis depends solely on the mitochondrial RNA polymerase [22,23]. Thus, metazoan primase-helicases harbors a primase module that has lost its priming activities. The similarities between the metazoan replicative mitochondrial DNA primase-helicase and the primase-helicase of bacteriophage T7 resulted in the name of TWINKLE (T7 gp4-like protein with intra-mitochondrial nucleoid localization) for this protein [24].

### 2.1. A T7-Like Replisome in Plant Organelles

In this review, we focus on the proteins from the model plant *Arabidopsis thaliana* as a representative of flowering plants. As their metazoan counterparts, plant organelles harbor enzymes related to the T7 replisome (Table 1). From the four enzymes involved in DNA replication in bacteriophage T7 and metazoan mitochondria, land plants have conserved three of them: (a) The primase-helicase, (b) the RNA polymerase, and (c) the single-stranded DNA binding protein (Table 1). The presence of these proteins suggests that plant mitochondrial DNA replication is executed in part by a mechanism that resembles the coordinated leading and lagging-strand replication model of bacteriophage T7 [22]. In this model, a central primase-helicase unwinds dsDNA in the 3’-5’direction followed by a processive DNA polymerase in the leading strand. The primase module of the primase-helicase uses the unwounded single-stranded regions to recognize a sequence to start the synthesis of very short ribonucleotides that are handed off to the active site of the lagging strand DNA polymerase. The single-stranded DNA regions generated during this trombone mechanism are coated by the single-stranded binding proteins [22,25].

#### 2.1.1. Plant Organellar Primase-Helicase (AtTwinkle)

**Figure 1:**
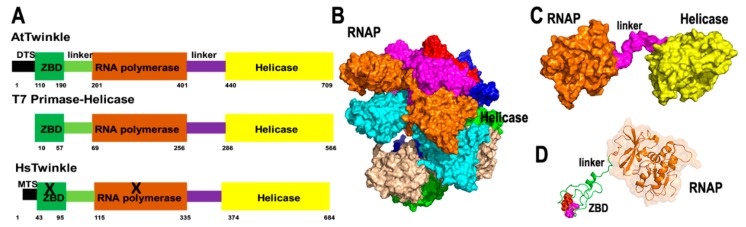
AtTwinkle is a homolog of bacteriophage T7 primase-helicase and mitochondrial Twinkle. (**A**) Schematic representation of the bifunctional T7 primase-helicase in comparison to AtTwinkle and human Twinkle. T7 primase-helicase and AtTwinkle contain the conserved motifs necessary for primase and helicase activities, whereas human Twinkle is inactive as a primase. (**B**) Homology model of AtTwinkle showing its RNA polymerase domain and helicase modules with basis on the crystal structure of the heptameric T7 primase-helicase [31]. (**C**) Close view of a monomeric module of the RNAP and helicase of AtTwinkle. (**D**) Close view of the primase module composed of the zinc binding domain (ZBD) and RNAP domain. The conserved cysteines that coordinate the zinc atom are colored in red and magenta.

Primase-helicases are the central component of replisomes [26,27]. These enzymes unwind double-stranded DNA segments using NTP hydrolysis for translocation and primer synthesis, using their helicase and primase modules, respectively [22,27]. The organellar primase-helicases in *A. thaliana* (dubbed AtTwinkle) is a 709 amino acid protein with mitochondria and chloroplast localization [28] (Figure 1A). AtTwinkle, as predicted for all plant primase-helicases, harbors both primase and helicase activities [28,29,30]. Structural studies of primase-helicase show that these enzymes assemble as heptamers or hexamers in which the helicase modules form a compact oligomeric ring to which the primase modules attach [31,32] (Figure 1B,C). The primase module of AtTwinkle contains six conserved motifs [30]. Motif I corresponds to the zinc binding domain (ZBD) necessary for template recognition, whereas regions II to VI assemble the RNA Polymerase domain (Figure 1D). In contrast to all previously characterized primase-helicases, AtTwinkle recognizes two cryptic nucleotides within the ssDNA template [29], a biochemical property that may reduce the length of the Okazaki fragments during plant mitochondrial replication. The helicase module of AtTwinkle shares high amino acid identity with the helicase module of the T7 primase-helicase and harbors the five conserved motifs [33], including a Walker motif necessary for nucleotide hydrolysis. The presence of an active AtTwinkle protein in Arabidopsis suggests the presence of a plant mitochondrial replisome in which a DNA polymerase replicates DNA following the unwinding of the double helix and exposing the leading-strand for continuous synthesis [34]. The primase activity suggests that a trailing DNA polymerase synthesizes the lagging-strand using primers synthesized by the primase module of AtTwinkle [29]. This model of coordinated leading and lagging strands occurs in bacteriophages T4 and T7, but not in mitochondria from metazoans and yeast [22,23,26]. Interestingly, Arabidopsis harbors a protein that contains the zinc finger and the RNA polymerase module of AtTwinkle dubbed AtTwinky [28]. This module by itself is functional in vitro [29]. An Arabidopsis insertional line in AtTwinkle shows no apparent phenotype, maybe because the T-DNA insertion occurs in an intron or because of redundant mechanisms for primer synthesis and DNA unwinding [34].

#### 2.1.2. Bacteriophage-Type Plant Organellar RNA Polymerases

**Figure 2:**
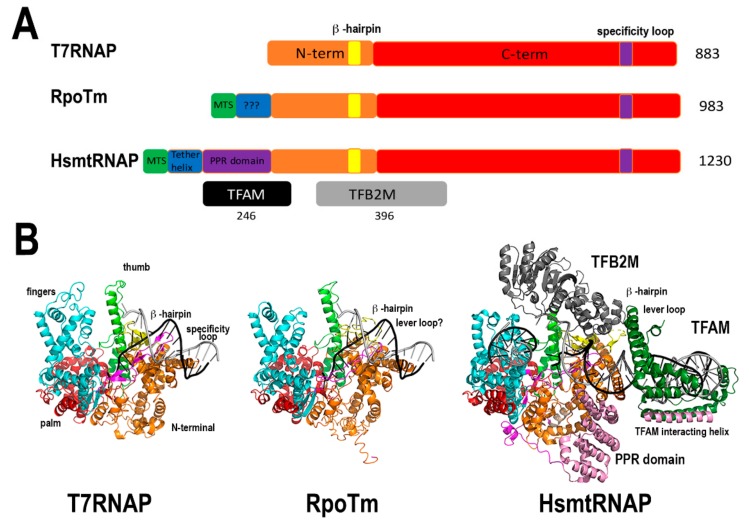
Bacteriophage-type plant organellar RNA polymerases. (**A**) Domain organization of bacteriophage-related RNAP. These enzymes share a C-terminal or polymerization domain that is divided into three subdomains: Fingers, palm, and thumb, and a N-terminal domain involved in promoter opening and RNA binding. The N-terminal domain is colored orange and the subdomains of the fingers, thumb, and palm of blue, green, and red, respectively. mtHsRNAP associates with two accessory subunits (TFB2M and TFAM) to open double-stranded DNA and contains a N-terminal pentatricopeptide repeat (PPR)-domain and a tether helix not present in plant mitochondrial RNAPs. (**B**) Structural model of the mtAtRNAP compared to bacteriophage T7RNAP and human mtRNAP during transcription initiation [40,53].

In yeast and metazoans mitochondria, transcription is carried out by a single RNA polymerase (mtRNA) homologous to T7 RNA polymerase [35]. In contrast to metazoans that harbor one nuclear-encoded mtRNAP, flowering plants encode three bacteriophage-type RNA polymerases [36,37]. One is localized into the mitochondria (RpoTm), one into the chloroplast (RpoTp), and the third one presents dual mitochondrial and plastid localization (RpoTmp). In Arabidopsis, RpoTm and RpoTp start transcription at a specific set of promoters. However, RpoTmp is unable to start transcription by itself [38]. These enzymes are closely related to bacteriophage T7 RNAP and due to sequence similarity are expected to fold into two conserved domains: An N-terminal domain, possibly involved in RNA binding and a C-terminal or polymerization domain. The C-terminal domain is structurally divided into three subdomains, dubbed palm, fingers, and thumb (Figure 2). Yeast and metazoan mitochondrial RNAPs are only active by themselves on supercoiled templates; on linearized templates, they need an associated transcription factor to start transcription [39,40]. Likewise, plant mitochondrial RNAPs are only active in supercoiled templates [36], suggesting that they also need an unidentified plant mitochondrial transcription factor for efficient promoter melting. Mitochondrial RNAPs from metazoans and yeast contains an N-terminal pentatricopeptide repeat (PPR) not present in plant mitochondrial RNAPs and T7 RNAP (Figure 2). Thus, plant mitochondrial RNAP are more compact than yeast and metazoan mitochondrial RNAPs.

In bacteriophage T7 and metazoan mitochondria, their RNAPs synthesize long RNA chains at defined sequences that mark their origins of replication [15,41,42,43]. It is unknown if plant mitochondrial RNAPs play a role in synthesizing RNA primers during mitochondrial or plastid replication. However, plant mitochondrial genomes are proposed to exist as a multitude of linear fragments, carrying only partial segments of their genome [44,45,46]. The presence of numerous promoter DNA sequences in plant mitochondria makes possible the existence of multiple initiation replication sites in mitochondrial DNA.

During metazoan mitochondrial DNA replication, the RNA primers generated by the mitochondrial RNA polymerase are removed by a specific set of nucleases. In humans, five different nucleases participate in this process [47,48,49,50,51,52]. From those enzymes, RNAse H1 plays a predominant role by degrading the RNA primer until it reaches few nucleotides. These last two to three ribonucleotides can be removed by the flap specific nucleases FEN1, DNA2, and MGME1 or by the selective 5′-3′ exonuclease EXOG [48,51]. Arabidopis encodes for three proteins highly homologous to RNase H1, dubbed AtRNH1A (At3g01410), AtRNH1B(At5g51080), and AtRNH1C (At1g24090) [47]. AtRNH1A is localized into the nucleus, whereas AtRNH1B and AtRNH1C are imported into mitochondria and chloroplasts, respectively. AtRNH1C prevents R-loop accumulation in chloroplast especially at highly transcribed regions and putative origins of replication [47]. AtRNH1C is involved in assuring genome stability in the chloroplast, suggesting the possibility that AtRNH1B may contribute to the removal of RNA primers in plant mitochondria.

#### 2.1.3. Plant Organellar Single-Stranded DNA Binding Proteins

**Figure 3:**
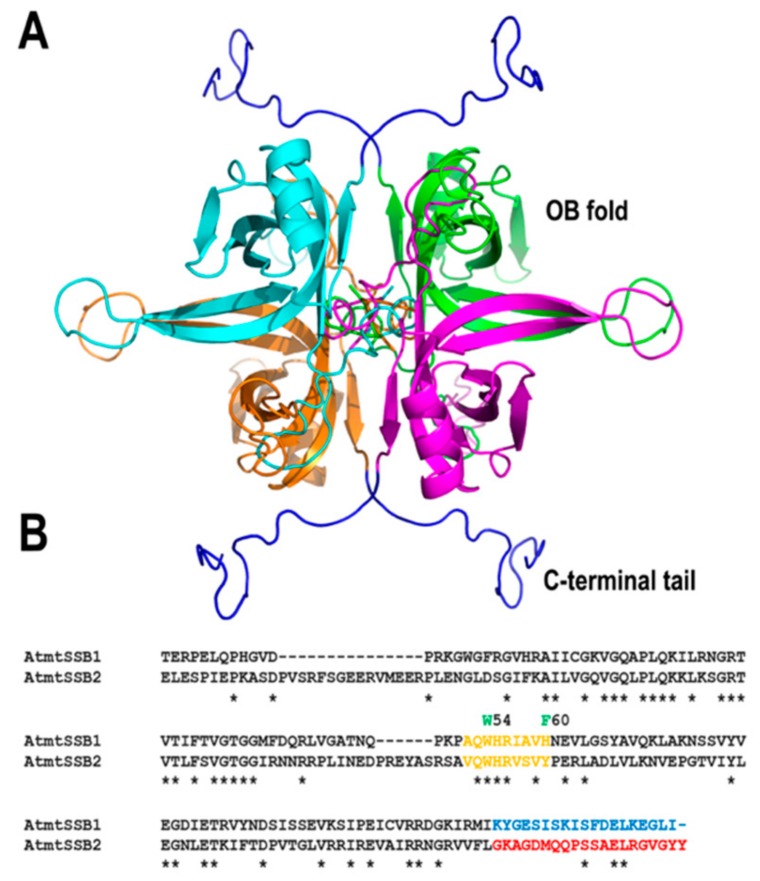
Homology model of tetrameric AtmtSSB1. **(A**) Homology model of AtmtSBB1 illustrating its oligonucleotide/oligosaccharide/binding (OB)-fold and an acid C-terminal tail. (**B**) An amino acid sequence alignment illustrates that the C-terminal tail of AtmtSSB2 is composed of two aromatic amino acids, whereas AtmtSSB1 is acidic.

All replisomes contain single-stranded DNA binding proteins (SSBs) that coat the lagging-strand DNA chain and exert a multitude of interactions with DNA polymerases, DNA helicases, and other proteins involved in DNA metabolism. Flowering plants encode for two canonical single-stranded DNA binding proteins that are targeted to mitochondria (AtmtSSB1 and AtmtSSB2) [54,55]. Like all SSBs, these proteins harbor an oligonucleotide/oligosaccharide/binding (OB)-fold domain and share a conserved set of aromatic amino acids that in other bacterial and mitochondrial SSBs are important for binding to single-stranded DNA. Among these amino acids, residues W54 and F60 that are determinant for binding to SSB in bacteria are conserved in AtmtSSB1 and AtmtSSB2 [56,57,58] (Figure 3). AtmtSSB1 assembles as a tetramer, binds single-stranded DNA in the nanomolar range, and interacts with plant mitochondrial DNA polymerases from Arabidopsis [59]. A recent proteomic analysis indicates that both AtmtSSB1 and AtmtSSB2 are highly abundant proteins, suggesting that a great portion of the mitochondrial single-stranded DNA is coated with them [55]. The last nine amino acids of *E. coli* SSB are responsible for mediating protein–protein interactions [60,61]. AtmtSSB1 contains a predominant acid tail while AtmtSSB2 harbors an aromatic tail (Figure 3), suggesting the possibility that both SSBs exert differential protein–protein interactions.

### 2.2. A Putative Recombination-Dependent Replication System in Plant Mitochondria

One of the main differences between plant and human mitochondrial genomes resides in the presence of highly abundant repeats of different lengths in plant mitochondria [62,63]. These repeats are classified by Gualberto and Newton as large repeats (>500 base pairs); intermediate-sized repeats (50–500 base pairs); and small repeats (<50 base pairs) [64,65]. Seminal studies deduced that the recombinogenic character at large repeats is responsible for plant mitochondrial DNA genomic configurations [62,66,67]. Thus, it is generally accepted that recombination at large repeats results in the presence of multiple mitochondrial genome conformations, whereas recombination at intermediate-size repeats are not as frequent [5,68]. The low-frequency recombination at intermediate-size repeats leads to changes in the stoichiometry of the mitochondrial genomes [69,70]. Finally, recombination at small repeats drives the apparition of new open reading frames associated with traits like cytoplasmic male sterility [71,72]. The notion that recombination is dependent on the length of the repeat is challenged by comparing new mitochondrial DNA sequences between domesticated and wild-type cultivars and by following the evolutionary history between species [73,74].

The recombinant character of the mitochondrial genome is reminiscent of bacteriophage T4 genome, which uses a recombination-dependent replication (RDR) mechanism [46,75]. Furthermore, seminal studies have shown the presence of linear molecules, head-to-tail concatemers, branched, and rosette-like structures during plant mitochondrial replication suggesting that free single-stranded DNA ends direct primer formation [45,46,76,77]. In contrast to metazoan mitochondria, plant mitochondria harbor a complete set of enzymes involved in HR. In bacteriophage, T4 RDR starts by coating of the single-stranded DNA by a recombinase dubbed UvsX, a protein homolog to bacterial RecA, or eukaryotic Rad51. As all recombinases, this protein uses ATP to catalyze the exchange of the single-stranded DNA into double-stranded DNA. This initial step creates a triple-stranded DNA region in which T4 DNA polymerase assembles to initiate replication. A replicative helicase loads onto the displaced DNA strand, this enzyme translocates in 5’ to 3’ direction, unwinding DNA, and generating a template for the trailing polymerase. The helicase associates with a primase that recognizes single-stranded sequences in the 3’-5’direction and generates primers used by a second DNA polymerase during replisome assembly. Although this system is relatively simple, it needs the presence of several mediator proteins that coordinate protein loading. In Arabidopsis mitochondria, several homologs to the battery of T4 enzymes involved in RDR are present, suggesting the possibility that RDR is a functional mechanism in plants (Table 2).

#### 2.2.1. AtRecA

**Figure 4:**
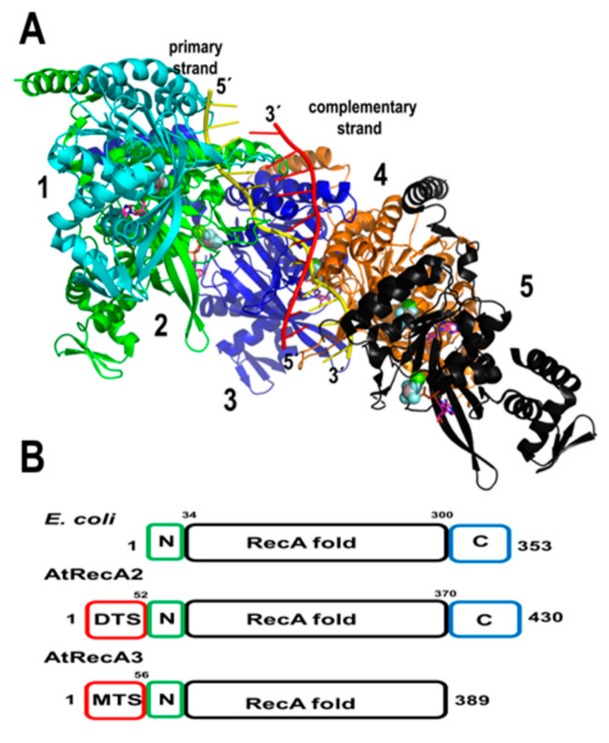
Structural conservation of plant and bacterial RecAs. (**A**) Crystal structure of the bacterial RecA postsynaptic nucleoprotein filament determined by Chen, Yang, and Pavletich [83]. Each of the five RecA monomers is individually colored and labeled with numbers. The search strand is colored in yellow and the complementary strand in red. The crystal structure comprises solely the RecA fold and the C-terminal domain is not present in the initial construct. (**B**) Domain organization of AtRecA2 and AtRecA3 in comparison to bacterial RecA. AtRecA3 lacks the C-terminal regulatory domain.

RecA and its homologs Rad51 and BRCA are the central components of homologous recombination. RecA is an archetypical bacterial recombinase that loads onto resected single-stranded DNA in an ATP-dependent reaction. It assembles a nucleic acid-protein filament that navigates the double-stranded genome in search of a homologous sequence, and when a region of homology is encountered, this filament perfectly pairs with its homologous partner (located within a dsDNA region) and generates a heteroduplex or D-loop intermediate [78]. HR by Rad51/RecA is abrogated in the presence of mismatches and bacterial RecA needs at least eight nucleotides of perfect complementarity to form a stable D-loop, although the efficiency of heteroduplex formation increases according to the length of the perfect complementarity [79,80,81]. In bacteria, the RecA monomer consists of a central or core domain of approximately 230 amino acids. This domain folds into a single β-sheet and six α-helices [82]. This core domain is flanked by N and C-terminal domains of approximately 30 and 60 amino acids, respectively [82]. The crystal structure of bacterial RecA–ssDNA filament illustrates how the RecA assembles onto ssDNA and how Watson–Crick pairing is assured during the homology search [83] (Figure 4A).

Unlike metazoan mitochondria that are devoid of RecA homologs, plant mitochondria harbor orthologues of the recombinase RecA/Rad51 gene family [69,84,85,86]. These proteins are conserved from algae to flowering plants. Genetic studies in *Physcomitrella* patens and *Arabidopsis* demonstrate the role of RecA in preventing illegitimate recombination events at small repeats in *P. patents* and intermediate-size repeats in Arabidopsis [69,86,87]. *A. thaliana* harbors three RecA genes. RecA1 is targeted to the chloroplast, RecA2 is targeted to plastids and mitochondria, whereas RecA3 is only targeted to mitochondria [69,88]. AtRecA1 is an essential gene, whereas AtRecA2 is only necessary after the seedling stage [69,86]. AtRecA2 and AtRecA3 share 53% and 41% amino acid identity with *E. coli* RecA, respectively. The latter suggests that HR in plant mitochondria may follow a mechanism similar to bacteria. Interestingly, AtRecA3 lacks the last 22 amino acids of its C-terminal domain in comparison to *E. coli* RecA. In bacteria, these residues have a highly acidic composition and a deletion of 17 amino acids is more efficient in displacing bacterial SSB from ssDNA, thus the C-terminal extension negatively modulates RecA activity [89] (Figure 4). Plants mutated in AtRecA3 are phenotypically normal. However, they are sensitive to genotoxic treatments [69]. The loss of RecA2 and RecA3 promotes rearrangements at intermediate-size repeats [86]. These repeats are not perfect and lead to homeologous recombinant products (illegitimate recombination products). The increase of illegitimate recombination products in the absence of AtRecA2 or AtRecA3 suggests that less stringent RecA-independent pathways take over in their absence. One possible pathway is the single-strand annealing recombination pathway (SSA) under the control of specialized SSBs with annealing capabilities as is the case in *Deinococcus radiodurans* [90]. Recent proteomic studies indicate that RecA2 is one of the most abundant DNA binding proteins in plant mitochondria [55].

#### 2.2.2. AtRecX

**Figure 5:**
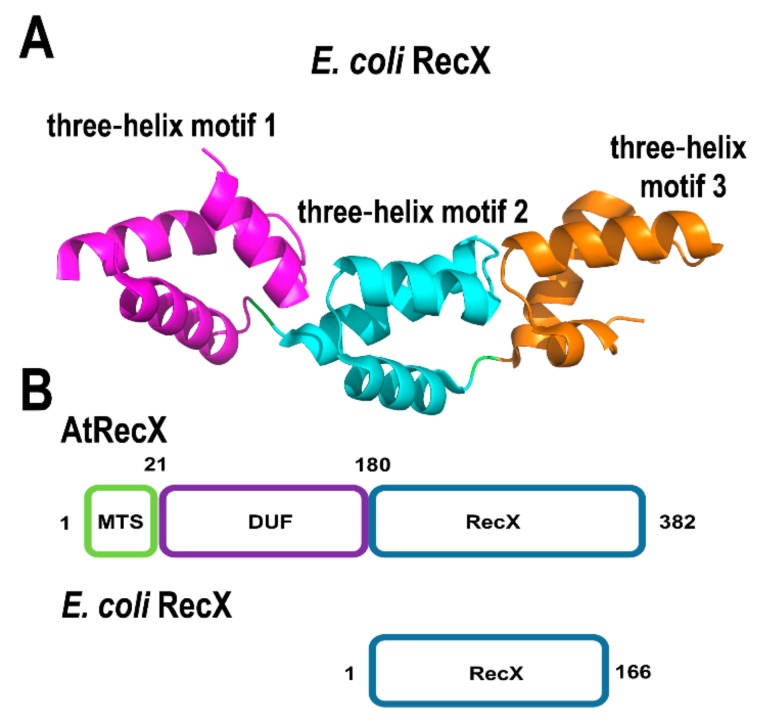
Structural organization of AtRecX. (**A**) Crystal structure of RecX from *E. coli* (PDB: 3c1d). RecX is composed of three repeats of a three-helix motifs, (**B**) modular organization of AtRecX in comparison to bacterial RecX. Plant RecX harbor a mitochondrial targeting sequence (MTS) and a N-terminal domain of unkown function. AtRecX share more than 30% amino acid identity with bacterial RecXs.

In bacteria, RecA can be inhibited by an interaction with a small protein (approximately 20 kDa) dubbed RecX [91]. RecX proteins bind to RecA monomers and DNA [92]. Bacterial RecX proteins are composed of nine α-helices that arrange into three three-helix bundles [93,94] (Figure 5A). RecX binds to RecA filaments promoting their dissociation from single-stranded DNA and impinging homologous recombination [95,96]. *A. thaliana* encodes for a gene of 382 amino acids, ortholog to bacterial RecX, with a predicted mitochondrial localization signal in its first 25 amino acids, a domain of unknown function and a C-terminal segment that presents 30% amino acid identity with *E. coli* RecX (Figure 5B). The presence of this RecX ortholog (AtRecX) suggests the possibility that RecA activities are subject to regulation in plants. The presence of three RecA genes in flowering plants also suggests that these proteins may be subject to a gradient of regulation by RecX in vivo. In the moss *Physcomitrella patens* RECX, overexpressing mutants exhibit increased recombination products at short dispersed repeats in mitochondria [97], suggesting that RecX modulates RecA activity and when RecA is not functionally active, less accurate DNA repair routes gain access to ssDNA with a concomitant appearance of illegitimate recombination products.

#### 2.2.3. Organellar DNA-Binding Proteins (ODBs)

**Figure 6:**
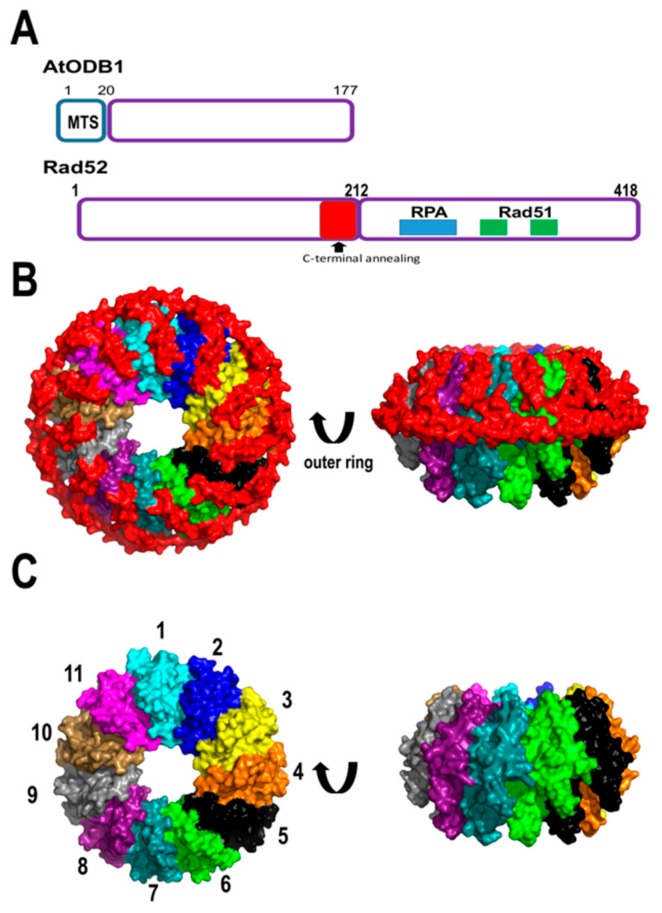
AtODB1 resembles human Rad52. (**A**) Structural domain organization of AtODB1 in comparison to human Rad52. AtODB1 lacks the C-terminal domain necessary to interact with RPA and Rad51; (**B**) crystal structure of the undecameric ring of human Rad52. The undecameric structure is stabilized by alpha-helix 5 that interacts with alpha-helix 1 of the neighbor molecule. Each subunit (residues 1 to 172 is individually colored) and the C-terminal residues (172 to 212) are colored in read. (**C**) Model of AtODB1 as a undecameric ring lacking alpha-helix 5 of human Rad52.

Upon the formation of single-stranded breaks, canonical SSBs bind to ssDNA blocking its acess to other binding proteins. In order for RecA to bind ssDNA, SSBs have to be removed from ssDNA. In bacteria, a protein named RecO (or its functional homolog in yeast, Rad52) interacts with the C-terminal tails of SSBs creating space for RecA binding [98]. Via proteomic studies, the Gualberto group identified that Arabidopsis contains two organellar DNA-binding proteins (ODBs), one located in the mitochondria (AtODB1) and the other in the chloroplast (AtODB2) [99]. AtODBs are homologous to Rad52 and the yeast mitochondrial nucleoid protein Mgm101 [100]. Mgm101 assembles an oligomeric ring structure and preferentially binds single-stranded DNA, suggesting a role in stabilizing and annealing DNA segments [101,102]. Likewise, Rad52 induces the displacement of human replication protein A (RPA) from ssDNA, anneals complementary ssDNA strands, and promotes strand exchange between ssDNA and dsDNA [103]. Thus, Rad52 promotes HR by displacing RPA, and promotes the coating of Rad51 by directing single-stranded annealing. Crystal structures of human Rad52 in complex with ssDNA depict this molecule as an undecameric ring in which two Rad52 oligomers could mediate HR in trans [104,105,106]. AtODB1 comprises 177 amino acids and shares extensive homology with the N-terminal domain of Rad52 (that contains the DNA binding and oligomerization regions). However, AtODB1 lacks a C-terminal domain containing the interacting motif for RPA and Rad51, that are involved in their displacement from ssDNA [107,108]. AtODB1 is 41 amino acids shorter than the construct of 212 amino acids used to crystallize human Rad52. Interestingly, the last 41 amino acids of human Rad52 folds into an alpha-helix (named helix 5) that intercalates with the first alpha-helix of the structure stabilizing the oligomeric assembly [104] (Figure 6).

Because of the reduced size of AtODBs, it is unknown if these proteins interact with SSBs from plant mitochondria like AtmtSSBs, AtWhirlies, AtRecA, or AtOSBs. Arabidopsis odb1 insertional mutants present no variation in phenotype, however upon genotoxic stress, they show inferior homologous recombination potential and increased microhomology-mediated end joining (MMEJ) [100]. This suggests that plant ODBs may function as mediator proteins that promote the annealing of plant RecAs onto single-stranded DNA. Recombinantly expressed plant ODB1 can anneal short DNA sequences [100]. The increase in MMEJ in plants lacking AtODB1 may be related to a role of this protein in a single-strand annealing recombination pathway, since human Rad52 proteins promote this route [109,110].

#### 2.2.4. AtRadA

**Figure 7:**
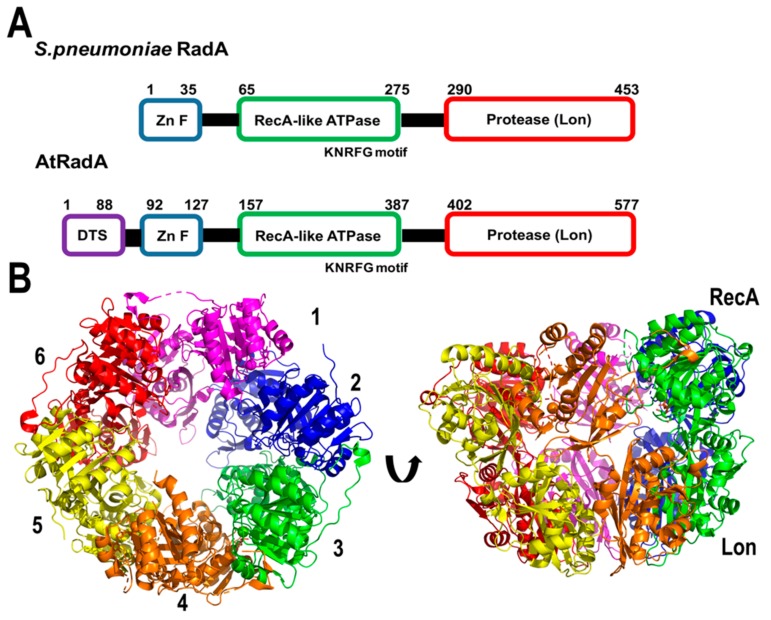
Plant RadA resembles the bacterial enzyme. (**A**) Structural organization of AtRadA in comparision to bacterial RadA. AtRadA shares 63% amino acid similarity with RadA from *S. pneumoniae* and complete amino acid identity in the catalytic amino acids. Bacterial RadA harbor a zinc finger (ZnF), a Rec-A like ATPase domain with a unique KNRFG motif, and a region homologous to the Lon protease. (**B**) Crystal structure of the Rec-A like ATPase and Lon protease domains of RadA from *S. pneumoniae* showing its resemblance to a hexameric helicase. The ZnF domain is not present in the crystal structure.

Bacterial RadA promotes single-stranded strand exchange similar to RecA, and was initially suggested to be orthologous to RecA [111]. Bacterial RadAs have a conserved domain organization composed of: (a) A putative zinc finger (ZnF), (b) a Rec-A like ATPase domain with a unique KNRFG motif, and (c) a region homologous to the Lon protease. Gualberto and Newton have identified the presence of a RadA-like gene in plant organelles [64] (At5g50340.1). This protein harbors a dual organellar targeting sequence in its first 88 amino acids and has 63% amino acid similarity with RadA from *Streptococcus pneumoniae* [112,113,114]. Bacterial Rad assembles as a hexameric ring, resembling the structural organization of replicative DnaB helicases [112] (Figure 7). Bacterial RadA interacts with RecA and unwinds dsDNA in the 3′-5′ direction. These biochemical properties suggest that RadA promotes the extension of ssDNA after RecA mediated homologous recombination, similar to the extension of bacterial origins of replication mediated by DnaB [112].

Because of the conserved domain organization of AtRadA, it is plausible that this protein is involved in a recombination-dependent replication mechanism. The appearance of multiple origins of replication in plant mitochondria by electron microscopy suggests the possibility that the unwinding ability of AtRadA is a key element for break-induced replication, by stabilizing a D-loop in synchrony with AtRecAs in which AtPolIs could be loaded. An interaction between RecA and RadA promotes D-loop extension in bacteria [115], suggesting that a similar mechanism could exist in plant mitochondria.

#### 2.2.5. AtRecG

DNA lesions like thymine-dimers or abasic sites, that potentially block replicative DNA helicases and DNA polymerases, are expected to be predominant in plant mitochondria. Thus, it is expected that plant mitochondria have developed mechanisms to avoid replication roadblocks that lead to replication fork collapse. Stalled replication forks can be resolved via the formation of four-strand Holliday junctions. In bacteria and bacteriophage T4, the helicases RecG and UvsW execute this process [75,116,117,118]. Bacterial RecGs are loaded in a stalled replication fork where they catalyze replication fork reversal by “pushing” a halted three-strand fork and convert this three-strand fork into a four-strand junction or Holliday junction [117,118,119,120]. The Holliday junction structure functions as a starting point for replication fork restart.

**Figure 8:**
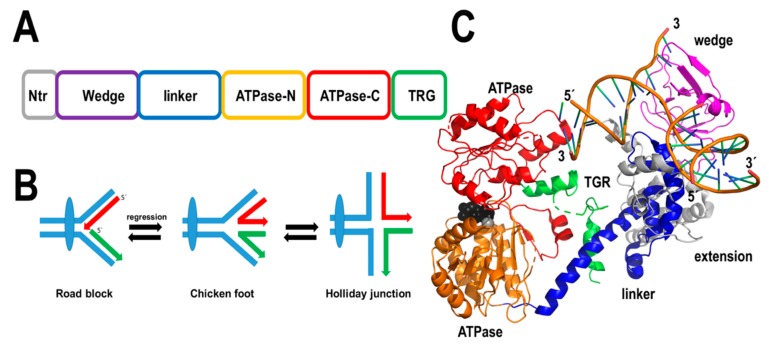
Plants harbor a RecG ortholog. (**A**) AtRecG presents the same domain organization of bacterial RecG, plus the addition of an N-terminal organellar targeting sequence. (**B**) RecG remodels halted replication forks by promoting fork regression (chicken foot structure) that is converted to a Holliday junction. (**C**) Crystal structure of *T. maritima* RecG illustrating its modular assembly.

Flowering plants encode a RecG homolog that is conserved from green algae [121]. In Arabidopsis this protein consists of 957 amino acids, from those residues its first 57 amino acids correspond to an organellar targeting sequence. AtRecG shares 34% amino acid identity with RecG from *Thermotoga maritima* and is expected to have a similar structure (Figure 8). Arabidopsis plants compromised in their RecG activity are prone to suffer recombination events at intermediate-size repeats and this phenomenon increases in plants deficient in AtRecA3 [121]. Although the precise role of AtRecG is unknown, this protein may be involved in the processing of Holliday junction structures and avoiding replication fork collapse or promoting DNA double-strand break repair.

### 2.3. Unique Proteins in Flowering Plant Mitochondria

Flowering plant mitochondria have unique proteins. These proteins include: (i) Replicative DNA polymerases solely encoded by protists and plants, (ii) a modified family of single-stranded binding proteins, dubbed organellar single-stranded DNA binding proteins (OSBs) in which their OB-fold suffered extensive modifications, (iii) an associated motif dubbed PDF that plays a role in binding to ssDNA, (iv) a protein that resembles Muts from bacteria, dubbed Msh1, that is only found in plants and corals, and (v) a distinctive family of proteins that belong to a family dubbed whirly (Table 3) [65,122,123,124,125,126,127,128]. Both Msh1 and whirlies are proposed to play a dual role in DNA metabolism and as sensor proteins via retrograde signaling from chloroplast-to-nucleus [129,130].

#### 2.3.1. Plant Organellar DNA Polymerases (POPs)

**Figure 9:**
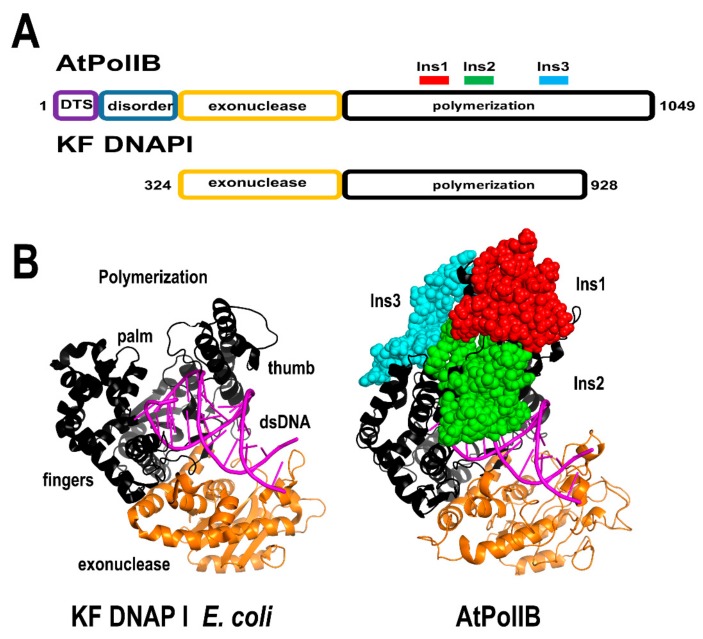
Structural comparison between AtPolIB and bacterial DNAPs. (**A**) Domain organization of both DNAPs. The polymerization domains are colored in black and the 3′-5′ exonuclease domains in orange. The unique amino acid insertions in AtPolIB in comparison to bacterial DNAPs I are depicted in a ball-stick representation and colored in red, green, and cyan. AtPolIs contain an N-terminal DTS and a disorder region not present in the structural model. (**B**) homology model of AtPolIBs with the crystal structures of the Klenow fragment from *E. coli* DNAP I. In both models, the dsDNA from Bacillus DNAP I is superimposed.

DNA polymerases in metazoan mitochondria are related to bacteriophage T-odd DNA polymerases [12,131]. Pioneering studies by the groups of Professors Sakaguchi and Sato revealed that plant organellar DNA polymerases have a different evolutionary history than phage and mitochondrial DNAPs from metazoans [122,123,124,125]. POPs belong to the family A of DNA polymerases; however, they did not evolve from bacteriophage T-odd DNAPs. Flowering plants harbor two paralogous POP genes with chloroplast and mitochondrial localization. In Arabidopsis, one POP is a high-fidelity DNAP (AtPolIA), whereas the other, AtPolIB, is a low-fidelity enzyme [132]. From a structural point of view, the most distinctive elements in POPs are the presence of three unique insertions in their polymerization domain, two of those insertions are located in the thumb subdomain (Ins1 and Ins2), whereas the third insertion is placed in the fingers subdomain [122,123,124,125]. Ins1 and Ins3 are involved in lyase, strand-displacement, and MMEJ activities [59,133,134] (Figure 9). AtPolIA and AtPolIB interact with AtTwinkle, and extend primers synthesized by its primase module [29,34]. The physical interaction between AtPolIs with AtTwinkle and AtSSB1 suggests the presence of a functional plant mitochondrial replisome [34]. Biochemical and functional evidence suggests that AtPolIA plays a predominant role in DNA replication, whereas the AtPolIB paralog plays a role in DNA repair [132,135,136]. The gene duplication event in POP evolution suggests a possible event of specialization. This situation resembles the presence of duplicated copies of the replicative DNA polymerase in Mycobacterium, in which one copy contributes to drug resistance because of its low nucleotide incorporation fidelity [137]. In this scenario, AtPolIB could be in the process of becoming a DNAP specialized in translesion synthesis or in other DNA repair pathways. Although AtPolIA and AtPolIB share more than 70% amino acid identity, a single amino acid change in homologous DNA polymerases provides translesion DNA synthesis capabilities [138].

#### 2.3.2. AtWhirlies

The most iconic family of single-stranded binding proteins in plant mitochondria is a family dubbed whirly. Whirlies are oligomeric proteins unique to plants. In contrast to the majority of organellar DNA binding proteins, whirlies are encoded in the nucleus and were initially identified as nuclear transcription factors [139]. Whirlies assemble as tetramers, however, upon binding to long-stretches of ssDNA they form a 24-mer assembly [140,141]. Arabidopsis harbors three members of the Whirly family, AtWhy2 localizes to mitochondria, and as a monomer is the most abundant DNA binding protein in plant mitochondria [55], whereas AtWhy1 and AtWhy3 translocate into chloroplasts [55,142]. T-insertional lines of Arabidopsis that knockout AtWhy1 and AtWhy3 accumulate DNA arrangements at microhomologous repeats in the chloroplast [143]. However, Arabidopsis plants devoid of AtWhy2 present a wild-type phenotype and do not accumulate MMEJ products in the absence of agents that induce DSBs [135,144], and show only a small increase in MMEJ products in presence of ciprofloxacin [135].

**Figure 10:**
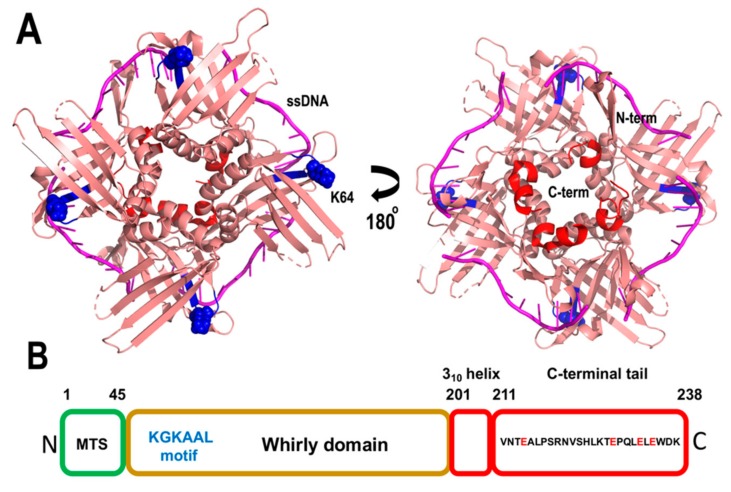
Structural organization of Whirlies. (**A**) Crystal structure of AtWhy2 (PDB ID: 4kop) with model ssDNA from Solanum whirly. The crystal structure represents residues 45 to 212. The second lysine of the KGKAAL motif is in a ball-stick representation. The C-terminal 3_10_ helix is in red. (**B**) Structural organization of AtWhy2. The disordered C-terminal tail is indicated in the diagram.

Whirly proteins bind ssDNA with nanomolar affinity and exhibit a novel protein fold in which each whirly monomer consists of two antiparallel beta sheets organized along two alpha-helices that resembles a whirligig [128,141]. The whirly domain comprises between 150 to 200 amino acids and contains an acidic/aromatic C-terminal end, that is disordered in crystal structures. The residues involved in ssDNA binding are distributed along the two antiparallel beta sheets and whirlies interact with ssDNA via hydrophobic residues and hydrogen bonds mediated by polar amino acids [140] (Figure 10). Whirlies harbor a conserved KGKAAL motif, located in the second beta strand of the first β-sheet, whose integrity is necessary for the 24-mer assembly [140]. Although mutations in this domain do not affect binding to short ssDNA segments, Arabidopsis complemented with a Why construct in which the second lysine of the KGKAAL motif is mutated to alanine are incompetent to reduce the appearance of microhomologies [140]. The latter suggests that the functional oligomeric state of Whirlies in vivo is a 24-mer. The solvent exposed localization of the unstructured C-terminal tail in whirlies suggests that they may mediate protein–protein interactions, analogous to bacterial SSB.

#### 2.3.3. Organellar Single-Stranded DNA Binding Proteins (OSBs)

**Figure 11:**
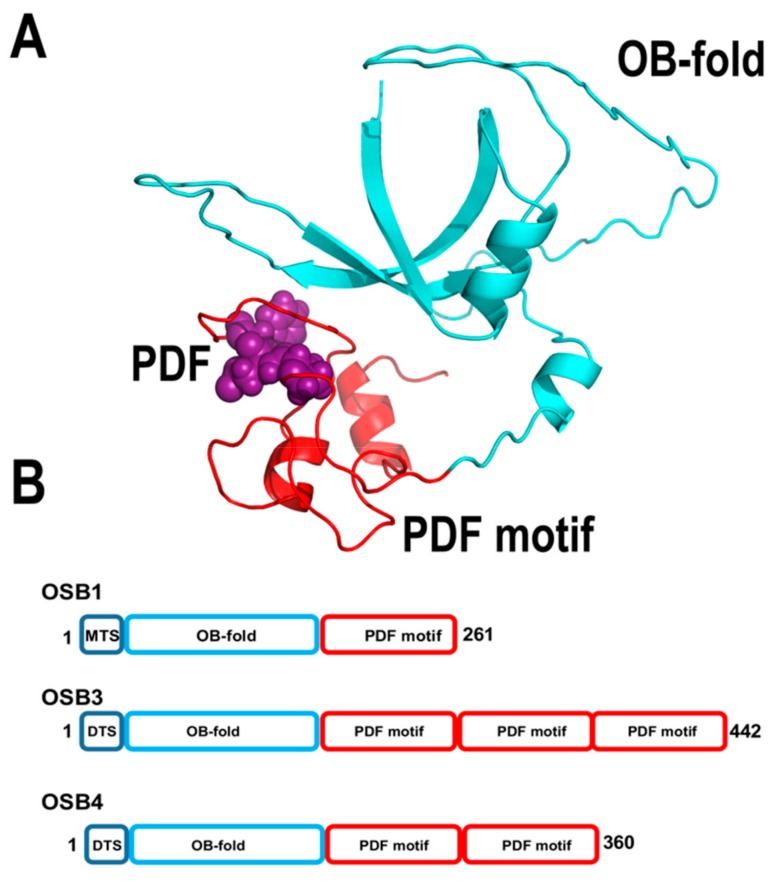
Structural organization of OSBs. (**A**) Structural model of AtOSB1 showing its predicted OB-fold and PDF motif domains. (**B**) Modular organization of mitochondrial OSBs in *Arabidopsis*. AtOSBs consist of an OB-like fold followed by one to three PDF motifs (54). Although AtOSB1 is depicted as a monomer, AtOSB2 in solution assembles as tetramer.

The groups of Gualberto and Imbault identified a unique family of single-stranded DNA binding proteins conserved from green algae to flowering plants [126]. These proteins harbor an N-terminal OB-fold domain linked to a motif of 50 amino acids dubbed PDF motif, because of a conserved signature of Pro, Asp, and Phe. Those researchers coined the name “Organellar Single-stranded DNA Binding proteins (OSB)” for members of this protein family. In OSBs, the PDF motif can be arranged as one or multiple copies (Figure 11). Arabidopsis contains four OSBs proteins, dubbed AtOSB1 to AtOSB4. AtOSB1and AtOSB2 are targeted exclusively to mitochondria and chloroplast, respectively, whereas AtOSB3 presents dual-target localization. Quantitative proteomic analysis showed that AtOSB4 and AtOSB3 are highly abundant proteins in mitochondria, whereas AtOSB1 is present at very low concentrations [55]. Remarkably, T-insertion lines of AtOSB1 generate homologous recombination products at repeats that are not commonly used [126].

AtOSB2 assembles as a tetramer and binds ssDNA with nanomolar affinity [59]. The PDF motif of AtOSB1 is sufficient for binding to ssDNA, whereas its OB-fold appears to have lost its ability to bind ssDNA [126]. AtOSB2 does not interact with AtPolIs, suggesting that in contrast to other single-stranded binding proteins, its role is not to avoid the formation of secondary structure elements that halt replicative DNA polymerases [59]. The high-affinity of AtOSBs for single-stranded DNA regions and their high abundance within mitochondrial DNA suggest that they coat single-stranded regions of DNA. This coating correlates with the increase of non-canonical homologous recombination products in plants lacking AtOSB1 [126].

#### 2.3.4. AtMhs1

George P. Rédei discovered that the CHLOROPLAST MUTATOR (chm) locus induces plant variegation and impaired fertility, and that both traits are inhered maternally [145,146]. The chm locus regulates the formation of rearrangements in plastids and mitochondria [147] and it encodes for a protein with resemblance to bacterial MutS, and therefore it was named Msh1 [65]. In bacteria, MutS and MutL are conserved elements of the DNA mismatch repair pathway. Within this pathway, MutS recognizes a mismatch and recruits the MutL endonuclease. Recognition of the mismatch correspondingly to the newly synthesized DNA chain is mediated by hemimethylation recognized by MutH [148]. The MSH1 gene is only present in corals and plants and is a multidomain protein harboring domains with homology to bacterial MutS and the GIY-YIG endonuclease [65,127,149,150]. Plants harboring deletions of this gene exhibit increased recombination frequencies at intermediate-size repeats. It is clear that Msh1 guards organellar genomes against aberrant or not frequent recombination events and the roles of Msh1 appear to be related to homeologous recombination suppression [5,68]. Thus, Msh1 resembles a minimal MutS/MutL complex, in which the GIY-YIG endonuclease may play the same role as that MutL endonuclease. In spite of its prevalent role in keeping a pristine plant mitochondrial genome, the only functional study of this protein comes from the characterization of its GIY-YIG domain. By itself this domain binds to branched DNA structures, however the individual domain is not active as an endonuclease [151]. The proposed role of Msh1 in supressing homeologous recombination resembles the role of MutS2 in Helicobacter pylori which harbors an Smr domain that is a non-specific endonuclease [152,153].

### 2.4. The Bacterial Gyrase, the Eukaryotic DNA Ligase, and the Archaeo-Eukaryotic PrimPol

#### 2.4.1. The Bacterial-Like Plant Organellar Gyrase

Topoisomerases are divided into two types, type I topoisomerases transiently introduce ssDNA breaks and type II transiently generate dsDNA breaks. DNA gyrase is a type II topoisomerase typically present in bacteria. This enzyme is a tetramer encoded by two subunits of the GyrA and GyrB proteins. Bacterial gyrases use ATP to introduce negative supercoils in DNA. Wall and coworkers discovered that flowering plants encode one gene for gyrA (At3g10690) and two functional genes of gyrB (At3g10270 and At5g04130) [154,155]. AtGyrA is targeted to mitochondria and chloroplast, whereas the product of At5g04130 is targeted to mitochondria and was dubbed AtmtGyrB [154]. Both AtGyrA and the two AtmtGyrBs have a clear cyanobacterial origin [154].

Structural studies of bacterial gyrases show the coordination between gyrA and gyrB that drives cleavage of the DNA strands, strand passage between subunits, and ligation [156,157,158]. Heterologously purified AtGyrA/AtmtGyrB present supercoiling activity [155] and the bacterial origin of the plant organellar AtGyrA/AtmtGyrB makes them a target for the development of new herbicides based on quinolones [155]. Ciprofloxacin, a quinolone drug, is commonly used to induce specific DSBs in plant organelles as the gyrase catalytic cycle is not completed [135,159]. However, bacterial DNA gyrases in complex with quinolone drugs pose a barrier for replication and transcription when bound to DNA and it is possible that the DBS results from the collision of replication forks [160]. As replication induces the formation of positive supercoils ahead of replication forks [161], the plant organellar DNA gyrase may control the formation of origins of replication and the rate of transcription.

#### 2.4.2. Nuclear DNA Ligase I Is Targed to Organelles

*Arabidopsis thaliana* encodes for three ATP dependent DNA ligases, dubbed DNA ligase I, IV, and VI. From these, DNA ligase I is located in the nucleus and mitochondria. DNA ligase IV is solely nuclear and DNA ligase VI is possibly targeted to both nucleus and chloroplast [162,163]. Thus, in flowering plants, DNA ligase I (At1g08130.1) is the only ligase known to be targeted to mitochondria [163]. DNA ligase I from Arabidopsis (AtDNAligI) shares 46% amino acid identity with DNA ligase I from humans and its mitochondrial targeting sequence is predicted to involve the first 53 amino acids [113]. The unique role of DNA ligase I in plants contrast with the situation in metazoans in which a specific DNA ligase, dubbed DNA ligase III, is the main DNA ligase in human mitochondria. Although this scenario appears to be specific to vertebrates and in lower eukaryotes, DNA ligase I is both a nuclear and a mitochondrial ligase [164,165]. DNA ligases I are structurally divided into three conserved domains: DNA binding, adenylation, and OB-fold. They also contain an N-terminal PCNA interaction motif, as the interaction between DNA ligase I and PCNA is crucial for efficient nick-sealing. Human DNA ligase I have a toroidal shape structure in which PCNA could be accommodated [166].

**Figure 12:**
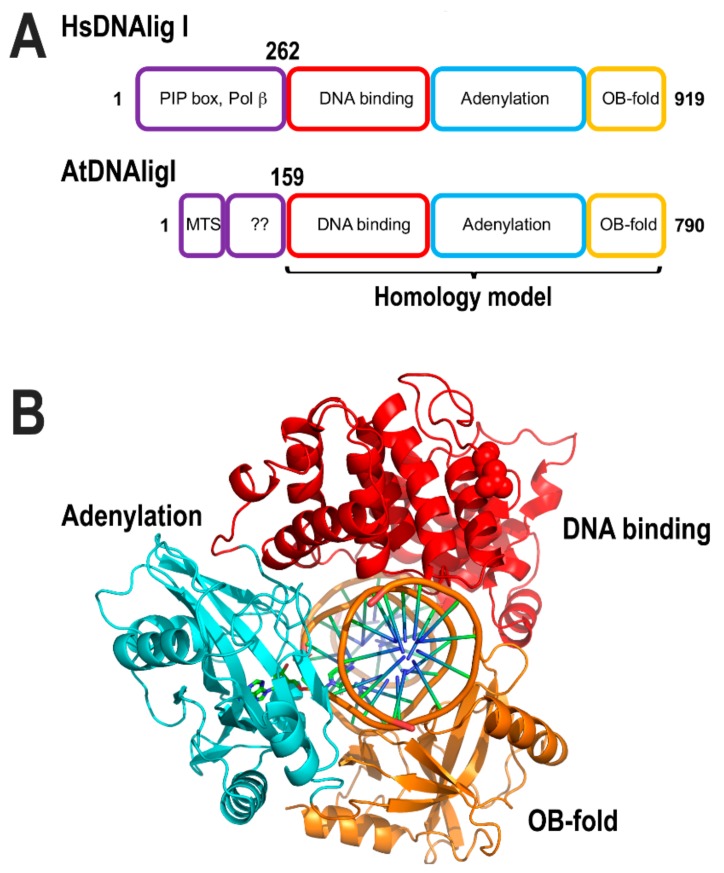
Structural comparison between HsDNAligI and AtDNligI. (**A**) AtDNAligI has a shorter N-terminal region. However, the core structure that harbors the DNA binding domain (red) the adenylation domain (cyan) and the OB-fold domain (orange) are conserved between both ligases. (**B**) Homology modeling of AtDNAlig I with basis on the crystal structure of human DNA ligase I (PDB ID: 1X9N).

The ligase active site is assembled between amino acids from the DNA binding and adenylation domains. Those domains harbors six conserved motifs (I, III, IIIa, IV, V, and VI) including the active site lysine, involved in the formation of the ligase–AMP intermediate [166,167]. As flowering plants appear to only have DNA ligase I in their mitochondria, this ligase is predicted to execute all nick sealing reactions. ATLIG1 is an essential gene and besides its role in DNA replication, it is involved in repairing single and DSBs [162]. A homology-based model of *A. thaliana* DNA ligase I using human DNA ligase I shows the predicted fold conservation between both proteins (Figure 12). The PCNA-interacting peptide (PIP box) motif, located at the N-terminal region of DNA ligases, is predicted to be absent in the mitochondrial isoform after its import into mitochondria (Figure 12). Although it is plausible that Arabidopsis DNA ligase I establishes a set of specific protein–protein interactions with protein partners in mitochondria, it is also possible that Arabidopsis DNA ligase I in mitochondria executes nick-sealing without the assistance of accessory proteins. Supporting this scenario, human mitochondrial DNA ligase III can be substituted for bacterial and viral ligases [168].

#### 2.4.3. Plant PrimPol

**Figure 13:**
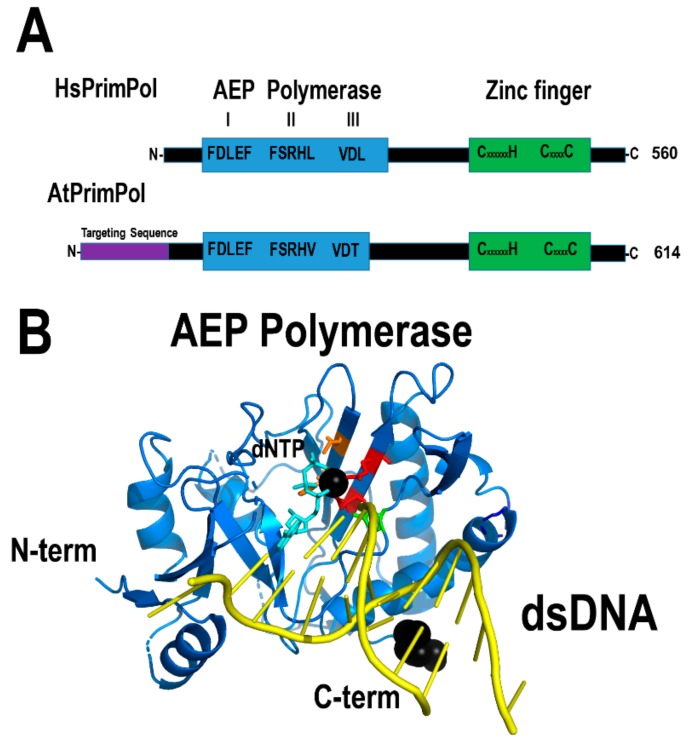
AtPrimPol resembles HsPrimPol. (**A**) Both AtPrimPol and HsPrimPol share a modular organization. AtPrimPol contains an N-terminal sequence for dual organellar targeting. (**B**) Structural model of the archaeo-eukaryotic primase (AEP) domain of AtPrimPol. The structural model was constructed with basis on the crystal structure of the AEP domain of HsPrimPol.

Three independent groups discovered that eukaryotic cells harbor a novel primase from the archaeo-eukaryotic primase (AEP) superfamily [169,170,171]. This enzyme is homologous to eukaryotic primases, but harbors both primase and polymerase activities in a single polypeptide and therefore it was dubbed PrimPol [169,170,171]. PrimPol contains independent AEP and zinc finger domains; the first domain is responsible for template-dependent nucleotide incorporation and the second domain provides a mechanism to recognize single-stranded DNA templates [170,172,173,174]. Human PrimPol localizes to the nucleus and mitochondria [170]. In human mitochondria, this enzyme is not involved in primer synthesizes during mitochondrial replication, but in negotiating DNA lesions by repriming and translesion DNA synthesis [169,175]. *Arabidopsis thaliana* harbors a PrimPol ortholog (AtPrimPol -At5g52800-). This enzyme is potentially a translesion synthesis DNA polymerase able of primer synthesis at specific single-stranded DNA sequences (Figure 13). This enzyme harbors localization signal for the nucleus, the mitochondria, and the chloroplast, suggesting that it may play a role in translesion DNA synthesis in each genome.

## 3. Known Unknowns in Plant Mitochondrial Replication

### 3.1. Mitochondrial DNA Replication Is Mosaic and Redundant

Plant mitochondrial DNA replication is carried out by mosaic and redundant elements (Table 1, Table 2 and Table 3). For instance, two DNA polymerases (AtPolIA and AtPolIB) are capable of executing DNA replication; at least three different processes may exist for DNA unwinding: (a) Direct unwinding by AtTwinkle, (b) direct unwinding by RadA, and (c) intrinsic unwinding by AtPolIs due to their strong strand-displacement activities; and five different processes (double stranded breaks, abortive transcription by mitochondrial RNA polymerases, and primer synthesis by AtTwinkle, AtTwinky, and AtPrimPol) could generate 3′-OHs needed to start replication. Thus, is not surprising that few genes involved in mitochondrial DNA replication are essential.

**Figure 14:**
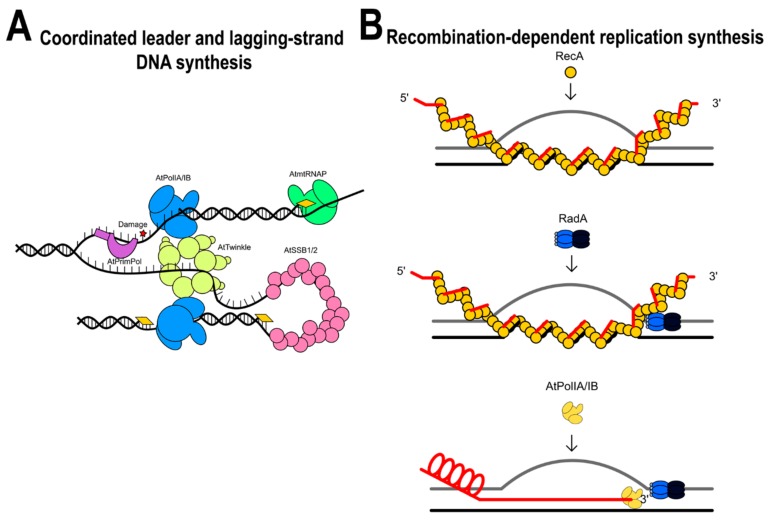
Putative models for DNA replication in plant mitochondria. (**A**) Leader and lagging-strand DNA synthesis. (**B**) Recombination-dependent replication systems in plant mitochondria.

In the coordinated leading and lagging-strand DNA synthesis model, an RNA polymerase synthesizes long RNA primers at unknown replication origins, AtTwinkle assembles at the single-stranded region, and these RNA primers are extended by a leading-strand AtPolI. AtTwinkle coordinates leader and lagging-strand synthesis by its primase activity. In the recombination-dependent replication system, a double-stranded break is resected and could be coated with AtRecAs. AtRecA would be responsible to find a homologous region in a double-stranded DNA segment. During AtRecA binding, the plant helicase AtRadA may bind to the single-stranded DNA assembling a replisome upon the interaction with AtPolIA or AtPolB (Figure 14).

In contrast to metazoan mitochondria, in which the four enzymes responsible for its replication are clearly related to enzymes from T-odd bacteriophages, plant mitochondria harbor enzymes with clear bacterial origin (DNA gyrase), proteins solely present in plant mitochondria (Msh1, OSBs, Why), and enzymes related to bacteriophages (AtTwinkle). This redundant and mosaic system may be responsible for the peculiarities present in plant mitochondrial genomes.

The study of DNA metabolism in plant mitochondria is in its infancy. We do not know how DNA replication in plant mitochondria starts, if plant mitochondria genomes need an origin of replication, and our knowledge of the physical interaction between the proteins involved in mitochondrial DNA metabolism is practically null. The classic view of the need of an origin of replication is given by the study of DNA replication in *E. coli*, where the initiator protein DnaA binds to specific sequences to drive replication initiation. In metazoan mitochondria, its RNA polymerase synthesizes RNA primers that function as primers for heavy and light chains, and it is generally accepted that yeast mitochondria start its replication at double-stranded breaks.

### 3.2. How Is the Accesibility to Single-Stranded DNA Regulated?

A recent proteomic analysis shows that AtRecA2, AtSSB1, AtSSB2, AtWhy2, AtOSB3, and AtOSB4 are among the most abundant proteins in plant mitochondria [55]. In solution, AtSSB1, AtWhy2, and AtOSB2 assemble as tetramers, although AtOSB2 readily form higher-order complexes (possible 8-mers or 16-mers) [59]. Surprisingly, AtWhy2 assembles as 24-mers in the presence of long segments of ssDNA (more than 7 Kbs) [140]. The carefull study by Fuchs and coworkers reveals that plant mitochondria contains approximately 140 tetramers of AtSSBs, 45 tetramers of AtOSB3 or AtOSB4, and 240 tetramers of AtWhy2 [59,128]. The abundance of AtWhy2 correlates with the fact that plants devoid of this protein accumulate DNA rearrangements mediated by microhomologous regions in the presence of agents that create DSBs [135,140]. Although no cellular studies using AtOSB2 or AtOSB3 have been carried out to date, AtOSB1 mutants accumulate homologous recombination products at repeats that are not commonly used [126]. Given that single-stranded regions of mitochondrial DNA are coated with AtSSB2s, AtWhy2, AtOSB2, and AtOSB3, it is unknown how these proteins are removed. A possible mechanism involves AtODB1, however AtODB1 lacks the C-terminal domain involved in protein–protein interactions. Thus, it is unknown if AtODB1 is able to displace ssDNA binding proteins like AtWhy2, AtSSBs, or AtOSBs from ssDNA or if AtSSBs interact with AtRecA2 to promote filament assembly.

### 3.3. Open Question in Plant Mitochondrial DNA Replication

It is puzzling how the open reading frames in plant mitochondria exhibit low substitution rates, while their non-coding regions are highly variable [6,9]. Mitochondrial DNA in land plants exists as linear molecules and it is proposed that neighboring DNA molecules can act as a template to avoid mutations [6]. If this is the case, it is unknown how the correct sequence is selected, given that plant mitochondrial DNA is not methylated. Furthermore, plant organellar DNA polymerases in Arabidopsis present a gradient of almost 10-fold in replication fidelity [134] and it is unknown if postraslational modification can affect their interaction with other proteins and their biochemical properties.

Several studies indicate the presence of non-homologous end joining (NHEJ) repair signatures in plant mitochondria. However, the key components of this route Artemis and Ku proteins are not targeted to plant mitochondria and the mechanisms by which a NHEJ-like route operate in plant mitochondria are unknown. Recent work using hybrid mitochondrial cell lines discovered that changes in the human epigenome are driven by modifications in the mitochondrial genome [176]. Does the highly recombinogenic nature of plant mitochondrial DNA confers an evolutionary advantage for flowering plants as a hub for adaptation?

## Figures and Tables

**Table 1:** Proteins related to bacteriophage T7 proteins present in plant mitochondria.

Enzyme	Phage T7	Human Mitochondria	Arabidopsis Organelles	Number	Localization
DNA Polymerase	T7 DNAP	DNAPγ	----	----	----
Helicase-Primase	Helicase-Primase	Helicase	AtTwinkle	At1g30680	Chloroplast and mitochondria
Primase	----	----	AtTwinky	At1g30660	?
Primase	T7 RNAP	mtRNAP	RpoTm	At1g68990	Mitochondria
----	----	----	RpoTmp	At5g15700	Chloroplast and mitochondria
SSB	SSB	mtSSB	mtAtSSB1	At4g11060	Chloroplast and mitochondria
----	----	----	mtAtSSB2	At3g18580	Mitochondria

**Table 2:** Plant mitochondrial proteins related to bacteriophage T4 recombination-dependent replication proteins.

Process or Enzyme	Phage T4	Bacteria	Arabidopsis Organelles	Acession Number	Localization
Annealing to ssDNA	UvsX	RecA	AtRecA2	At2g19490	Chloroplast and mitochondria
			AtRecA3	At3g10140	Mitochondria
Suppress Rec-A Annealing	----	RecX	AtRecX	At3g13226.1	?
Mediator	UvsY	RecFOR	AtODB1	At1g71310	Mitochondria and the nucleus
Helicase	Dda?	RadA	AtRadA	At5g50340	Chloroplast and mitochondria
Branch Migration Remodeling	UvsW	RecG	AtRecG1	At2g01440.1	RecG1

**Table 3:** Unique proteins involved in DNA metabolism in flowering plant mitochondria.

Arabidopsis Organeles	Acession Number	Localization
AtPolIA	At1g50840	Mitochondria and chloroplasts
AtPolIB	At1g30680	Mitochondria
AtWhy2	At1g71260	Mitochondria
AtOSB1	At1g47720	Mitochondria
AtOSB3	At5g44785	Mitochondria and chloroplasts
AtOSB4	At1g31010	Mitochondria, chloroplasts
AtMsh1	At3g24320.1	Nuclear, mitochondria and chloroplasts
